# Estrogen receptor α (ERα) mediates 17β-estradiol (E2)-activated expression of HBO1

**DOI:** 10.1186/1756-9966-29-140

**Published:** 2010-11-01

**Authors:** Wen-zhong Wang, Hai-ou Liu, Yi-hong Wu, Yi Hong, Jun-wu Yang, Ye-heng Liu, Wei-bin Wu, Lei Zhou, Lin-lin Sun, Jie-jie Xu, Xiao-jing Yun, Jian-xin Gu

**Affiliations:** 1 Department of Biochemistry and Molecular Biology, Shanghai Medical College of Fudan University, Shanghai, 200032, China; 2 Institutes of Biomedical Science of Fudan University, Shanghai, 200032, China; 3 Institute of Nautical Medicine, Nantong University, Nantong, 226001, China

## Abstract

**Background:**

HBO1 (histone acetyltransferase binding to ORC1) is a histone acetyltransferase (HAT) which could exert oncogenic function in breast cancer. However, the biological role and underlying mechanism of HBO1 in breast cancer remains largely unknown. In the current study, we aimed to investigate the role of HBO1 in breast cancer and uncover the underlying molecular mechanism.

**Methods:**

Immunohistochemistry was applied to detect HBO1 protein expression in breast cancer specimens (n = 112). The expression of protein level was scored by integral optical density (IOD) for further statistical analyses using SPSS. Real-time PCR was used to simultaneously measure mRNA levels of HBO1. The HBO1 protein expression in breast cancer cells was confirmed by western blot.

**Results:**

HBO1 was highly expressed in breast cancer tissues and significantly correlated with estrogen receptor α (ERα) (p < 0.001) and progestational hormone (PR) (p = 0.002). HBO1 protein level also correlated positively with histology grade in ERα positive tumors (p = 0.016) rather than ERα negative tumors. 17β-estradiol (E2) could upregulate HBO1 gene expression which was significantly inhibited by ICI 182,780 or ERα RNAi. E2-increased HBO1 protein expression was significantly suppressed by treatment with inhibitor of MEK1/2 (U0126) in T47 D and MCF-7 cells.

**Conclusions:**

HBO1 was an important downstream molecule of ERα, and ERK1/2 signaling pathway may involved in the expression of HBO1 increased by E2.

## Background

Breast cancer is a major public health issue, with more than one million new cases observed around the world in 2002 [1]. The pathogenesis of breast cancer is quite complex. Lifetime exposure to estrogen is reported to be associated with women's risk for breast cancer and the biological actions of estrogens are mediated primarily by ERα which belongs to the nuclear receptor superfamily, a family of ligand-regulated transcription factors [2-4]. ERα, which promotes cell growth, metastasis and also mediates resistance to apoptosis, plays a key role in progression of breast cancer [5,6].

HBO1 (histone acetyltransferase binding to ORC1), also named MYST2, belongs to the MYST family which is characterized by a highly conserved C2HC zinc finger and a putative histone acetyltransferase domain. The role of HBO1 in cancer remains unclear, although its expression has been reported in testicular germ cell tumors, breast adenocarcinomas, and ovarian serous carcinomas [7]. Recent investigations have revealed that over-expression of HBO1 dramatically enhances the anchorage-independent growth of both MCF7 and SKBR3 breast cancer cells [8]. Furthermore, it also functions as a transcriptional coactivator for hormone receptors including ERα and PR [9], leading to consideration of this protein as a carcinogenetic factor.

To elucidate the role of HBO1 in breast cancer, 112 breast cancer specimens were investigated using immunohistochemistry with an anti-HBO1 antibody. We found that HBO1 was notably increased in breast cancer. E2-upregulated HBO1 expression could be inhibited by ICI 182,780 or ERα RNAi in breast cancer cells. Furthermore, we also showed that ERK1/2 signaling pathway was involved in the expression of HBO1 increased by E2.

## Methods

### Materials

Dulbecco's modified Eagle's medium (DMEM), phenol red-free DMEM (PR-free DMEM), U0126 and 17β-estradiol (E2) were purchased from Sigma. Lipofectamine 2000, Trizol Reagent and fetal bovine serum (FBS) were purchased from Invitrogen. Charcoal-stripped fetal bovine serum (CS-FBS) was purchased from Biowest. PVDF membrane, leupeptin, aprotinin, phenylmethylsulfonyl fluoride (PMSF) and X-tremeGENE siRNA Transfection Reagent were purchased from Roche. RNA PCR Kit (AMV) Ver.3.0 was purchased from TaKaRa. Polinl-2-plus kit was a product of GBI. Anti-phospho-ERK1/2 (Thr202/Tyr204) and anti-ERK1/2 antibodies were purchased from Cell Signaling Technology. ERα siRNA, mouse monoclonal anti-ERα, anti-GAPDH, horseradish peroxidase (HRP)-conjugated goat anti-rabbit and HRP-conjugated goat anti-mouse IgG secondary antibodies were from Santa Cruz Biotechnology. Rabbit polyclonal anti-HBO1 antibody (Catalog No: 13751-1-AP) was purchased from Protein Tech Group, Inc. The enhanced chemiluminescence (ECL) assay kit was purchased from Tiangen. Dual-luciferase reporter assay system was bought from Promega. ICI 182,780 was purchased from Tocris Bioscience.

### Cell culture and transfection

Human MCF-7, T47 D, MDA-MB-453 and MDA-MB-435 breast cancer cells were obtained from the Cell Bank of Type Culture Collection of Chinese Academy of Sciences. MCF-7 cells were cultured in DMEM supplemented with 10% fetal calf serum (FBS), 10 ug/ml of insulin, 100 U/ml of penicillin and 50 ug/ml of streptomycin. T47 D cells were maintained in DMEM supplemented with 10% FBS, 100 U/ml of penicillin and 50 ug/ml streptomycin. SiRNA was transfected with X-tremeGENE siRNA Transfection Reagent according to the manufacture's instructions.

### Western blot analyses

Proteins were detected by western blot analysis as described previously [10]. The cells were lysed by lysis buffer, separated in 10% SDS-PAGE and then transferred to a PVDF membrane. The membrane was incubated with primary antibody followed by incubation with horseradish peroxidase-conjugated secondary antibody. Then the membrane was developed using the ECL detection system.

### Tissue samples

112 primary breast cancer specimens were consecutively obtained from pathological archives of Huashan Hospital, Fudan University from 2005 to 2008. There was no any bias for selection. Tissues were formalin-fixed and paraffin-embedded for histopathologic diagnosis and immunohistochemical study. The malignancy degree of tumor was scored according to the Scarff-Bloom-Richardson system.

### Immunohistochemistry (IHC)

Serial sections (5 μm thick) were mounted on glass slides coated with 10% polylysine. Sections were dewaxed in xylene and rehydrated in graded ethanol. Endogenous peroxidase activity was blocked by immersion in 0.3% methanolic peroxide for 30 minutes. Next, sections were microwaved in citrate buffer for antigen retrieval. Rabbit polyclonal anti-HBO1 (1:100 dilutions) was used as primary antibodies. Staining was completed with Polink-2-plus kit, in accordance with the manufacturer's instructions. Color reaction product was developed with diaminobenzidine. All sections were counterstained with hematoxylin. Two pathologists separately blinded to the clinical outcome of the patients evaluated all samples. The immunoreactivity intensity was evaluated as 0 (absent); 1 (weak nuclear staining); 2 (moderate nuclear staining of intermediate level); 3 (more coarse nuclear staining). Positive cells were quantified as a percentage of the total number of tumor cells with observation of 1000 cells in 5 high power field (×400), and assigned to one of five categories: 0: < 5%, 1: 5-25%, 2: 26-50%, 3: 51-75% and 4: > 75%. HBO1 expression was scored semi-quantitatively using the Remmele-score (immunoreactive score (IS) = score of percentage of positive tumor cells × score of staining intensity). Then the slide of IS > 3 was classified as a positive case [11]. Reproducibility of the scoring method used by both observers was greater than 95%.

### Total RNA extraction and real-time PCR

Total mRNA samples of T47 D and MCF-7 breast cancer cells were extracted using trizol reagent according to the manufacturer's instructions. One microgram of total RNA extracted from the cells was subjected to reverse transcription (RT). The RT and real-time PCR were performed by using TaKaRa RNA PCR Kit (AMV) Ver.3.0 and SYBR Premix Ex Taq II according to the manual respectively. Primers used for real-time PCR were as follows: HBO1-F 5'-GATGCCCACTGTATCATAACC-3' and HBO1-R 5'-TTCTTCCTGAGTTCAGCCACT-3'; GAPDH-F 5'-GGCTGAGAACGGGAAGCTTGTCAT-3' and GAPDH-R 5'-CAGCCTTCTCCATGGTGGTGAAGA-3'. Real-time PCR was performed using 7500 multicolor real-time PCR detection system (ABI) with the following cycling conditions: (i) 30s at 95°C and (ii) 40 cycles, with 1 cycle consisting of 5s at 95°C, 34s at 60°C. GAPDH was employed as an internal control under the same experimental conditions. Data were analyzed by using 7500 software (ABI). The values were obtained through normalizing to GAPDH copies.

### Statistical analysis

Statistical data analyses were performed using SPSS 11.5 statistical software package. The relationship between protein levels and different clinical and pathological features were explored using cross tabulation and Pearson's x^2^. P values less than 0.05 were selected.

## Results

### HBO1 protein level correlates positively with ERα

**Figure 1:**
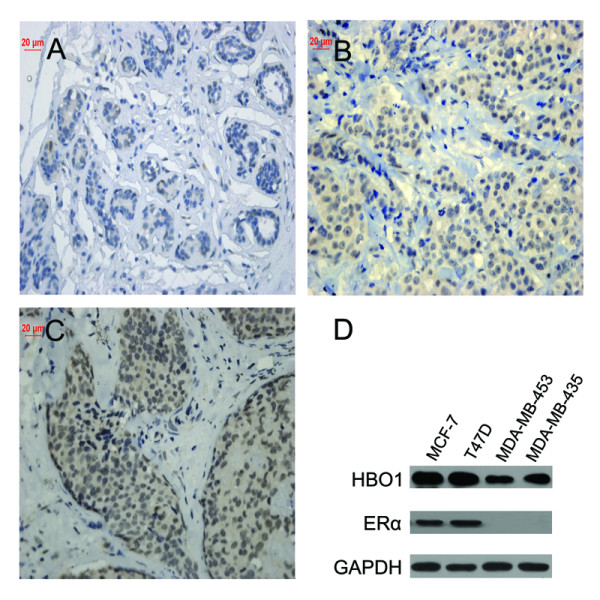
**Expression of HBO1 in human breast cancer**. (A) Immunohistochemical staining for HBO1 in benign breast epithelial tissues (magnification: A, 400×). (B) Moderately differentiated breast cancer tissues (brown staining) (magnification: B, 400×). (C) Poorly differentiated breast cancer tissues (brown staining) (magnification: C, 400×). (D) HBO1 protein level in several breast cancer cell lines based on the western blot results.

In order to explore the biological role of HBO1 in human breast cancer *in vivo*, we examined the expression of HBO1 in tumor samples of primary breast cancer (n = 112) by IHC analysis. We found that HBO1 which was predominantly detected in nucleus (Figure 1B-C) was highly expressed in breast cancer tissues (Table 1) and significantly correlated with ERα (p < 0.001) and PR (p = 0.002) (Table 2). Further statistical analysis revealed HBO1 protein level correlated positively with histology grade in ERα positive tumors (p = 0.016) rather than ERα negative tumors (Table 2). For benign breast tissues, low HBO1 immunoreactivity was observed and epithelial cells displayed a minimal granular staining (Figure 1A). Moderately and poorly differentiated breast cancer tissues showed intense HBO1 staining (Figure 1B-C). To further study the relationship between HBO1 and ERα, we examined HBO1 expression level in several breast cancer cell lines by western blot, which showed that ERα positive breast cancer cell lines exhibited higher HBO1 protein than ERα negative breast cancer cell lines (Figure 1D).

**Table 1:** Clinical and pathological characteristics of patients

characteristic	value
Tumor grade(n[%])	

I	45 [40.2%]

II/III	67 [59.8%]

Ki67 status(n[%])	

negative	50 [44.6%]

positive	62 [55.4%]

Estrogen receptor α status(n[%])	

negative	39 [34.8%]

positive	73 [65.2%]

Progesterone receptor status(n[%])	

negative	60 [53.6%]

positive	52 [46.4%]

P53 status(n[%])	

Negative	62 [55.4%]

Positive	50 [44.6%]

HBO1 status(n[%])	

negative	48 [42.9%]

positive	64 [57.1%]

Histology Grade (n[%])	

G1	32 [28.6%]

G2/G3	80 [71.4%]

**Table 2:** Expression of HBO1 in relation to the clinical and pathological characteristics of patients

clinical feature	total	HBO1 expression		*P*-value
		**negative**	**positive**	

Tumor grade				

I	45	21	24	0.610

II/III	67	28	39	

Ki67				

negative	50	23	27	0.550

positive	62	25	37	

Estrogen receptor				

negative	39	26	13	< 0.001**

positive	73	22	51	

Progesterone receptor				

negative	60	34	26	0.002**

positive	52	14	38	

P53				

negative	62	23	39	0.170

positive	50	25	25	

Histology Grade				

G1	32	17	15	0.165

G2/G3	80	31	49	

among ER positive tumors Histology Grade				

G1	25	12	13	0.016*

G2/G3	48	10	38	

among ER negative tumors Histology Grade				

G1	8	5	3	0.779

G2/G3	31	21	10	

*P < 0.05 **P < 0.01

### E2 induces HBO1 expression in breast cancer cells

**Figure 2:**
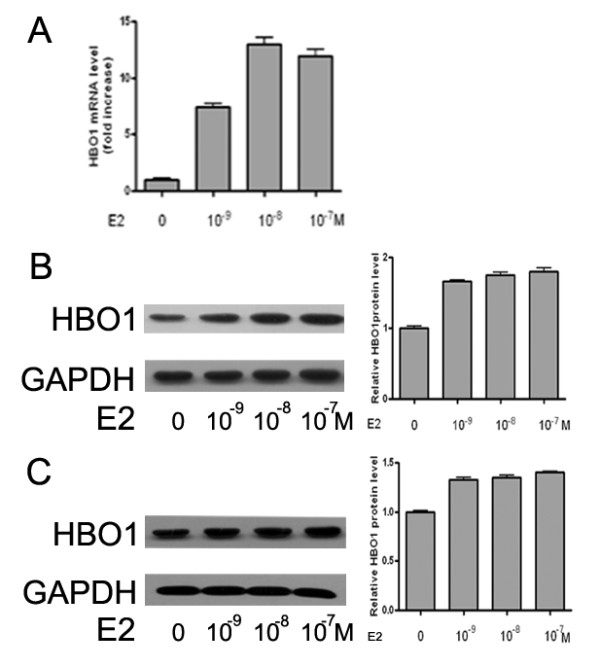
**E2 induces HBO1 expression in breast cancer Cells**. (A) T47 D Cells were treated with E2 (10^-9^, 10^-8^, 10^-7 ^M) for 24 hours and mRNA level was quantified by real-time PCR analysis. (B) T47 D cell were treated with E2 (10^-9^, 10^-8^, 10^-7 ^M) for 24 hours and western blot. Results quantitated by densitometric analysis were representative of three repeated experiments. (C) MCF-7 cells were treated with E2 (10^-9^, 10^-8^, 10^-7 ^M) for 24 hours and western blot. Results quantitated by densitometric analysis were representative of three repeated experiments.

In order to further investigate the relationship between ERα and HBO1, we treated breast cancer cells with 17β-estradiol (E2). Quantitative real-time PCR was used to determine the effect of E2 on the mRNA level of HBO1. With the dose of E2 increasing from 10^-9 ^to 10^-7 ^M, the mRNA level of HBO1 gradually increased and reached a plateau at 10^-8 ^M in T47 D cells (Figure 2A). Hereby, 10^-8 ^M of E2 was applied for all subsequent experiments. Western blot was also performed to confirm that E2 could upregulate HBO1 protein expression in T47 D (Figure 2B) and MCF-7 cells (Figure 2C). Taken together, these results demonstrated that E2 increased expression of HBO1 at transcriptional level.

### E2-induced HBO1 expression is inhibited by ICI 182,780 or ERα RNAi

**Figure 3:**
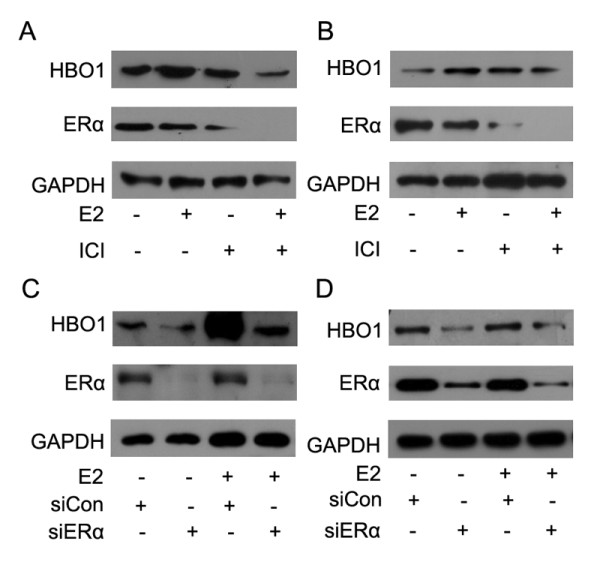
**E2-induced HBO1 expression is inhibited by ICI 182,780 or ERα RNAi**. (A) T47 D cells were treated with E2 (10^-8 ^M), ICI 182,780 (1 uM), or E2 (10^-8 ^M) plus ICI 182,780 (1 uM) for 24 hours. Then total cell lysates were processed for western blot analysis as described in Methods. GAPDH was used as an internal control. (B) MCF-7 cells were treated with E2 (10^-8 ^M), ICI 182,780 (1 uM), or E2 (10^-8 ^M) plus ICI 182,780 (1 uM) for 24 hours. Then total cell lysates were processed for western blot analysis as described in Methods. GAPDH was used as an internal control. (C) T47 D cells were transiently transfected with scrambled siRNA or ERα siRNA. 48 h later, total cell lysates were processed for western blot analysis as described in Methods. (D) MCF-7 cells were transiently transfected with scrambled siRNA or ERα siRNA. 48 h later, total cell lysates were processed for western blot analysis as described in Methods.

To further study the role of ERα in HBO1 expression, ICI 182,780 was further applied in T47 D cells (Figure 3A) and MCF-7 cells (Figure 3B) to block the effect of E2. As shown in Figure 3A, E2 increased HBO1 protein expression (lane 2), which was significantly reduced by ICI 182,780 (lane 4). Similar results were obtained in MCF-7 cells (Figure 3B). ICI 182,780 was reported to act by binding ERα, causing disassociation of ERα associated proteins and resulting in impaired receptor dimerisation and increased receptor degradation [12,13]. As expected, ERα expression was decreased by ICI 182,780 (Figure 3A and 3B). These results indicated that E2-induced HBO1 upregulation could be inhibited by ICI 182,780. In order to figure out the role of ERα in E2-induced HBO1 upregulation, ERα siRNA was transfected into T47 D and MCF7 cells to knockdown ERα. We observed that E2-induced HBO1 upregulation was inhibited by ERα siRNA (lane 4), indicating that HBO1 might be a downstream signaling molecule for ERα.

### ERK1/2 signaling pathway was involved in the expression of HBO1 increased by E2

**Figure 4:**
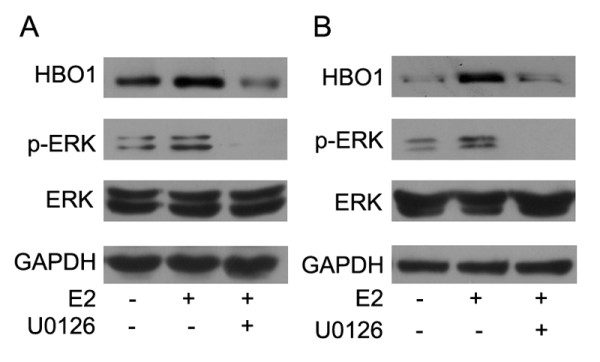
**E2 enhances HBO1 expression through ERK1/2 signaling pathways**. (A) Serum-starved T47 D cells were treated with E2 (10^-8 ^M), or U0126 (10 uM) plus E2 (10^-8 ^M) for 24 hours. Then equal amounts of protein (lysates) were subjected to SDS-PAGE. Western blot was performed using the Anti-phospho-ERK1/2 (Thr202/Tyr204), anti-HBO1 and anti-ERK1/2 antibodies. GAPDH was used as an internal control. (B) Serum-starved MCF-7 cells were treated with E2 (10^-8 ^M), or U0126 (10 uM) plus E2 (10^-8 ^M) for 24 hours. Western blot was performed as described in (A).

Using TRSEARCH software based on the sequence database, the promoter region of the HBO1 gene has no putative binding sites for ERα (data not shown). And previous studies have shown that steroidal estrogens are able to rapidly and transiently activate the ERK1/2 pathway [13], so we examined whether the ERK1/2 pathway was involved in the effect of E2 on HBO1 expression in breast cancer cells. As shown in Figure 4, E2-increased HBO1 protein expression was significantly suppressed by treatment with inhibitor of MEK1/2 (U0126) in T47 D (Figure 4A) and MCF-7 (Figure 4B) cells as analyzed by western blot. These results indicated that ERK1/2 signaling pathway was involved in the E2-induced HBO1 upregulation in breast cancer cells.

## Discussion

HBO1 is a potential oncogene which maps to17q21.3, a region where frequent allelic gains are found in breast cancers and this amplification is associated with a poor prognosis of clinical outcome [14-16]. Previous studies demonstrated over-expression of HBO1 dramatically enhanced the anchorage-independent growth of both MCF7 and SKBR3 breast cancer cells while depletion of HBO1 reduced the rate of DNA synthesis, the amount of MCM complex bound to chromatin, and progression through S phase. HBO1 has also been shown to enhance transcription mediated by steroid receptors including ERα and PR [9]. However, little is known about the role of HBO1 in breast cancer and the underlying molecular mechanism.

In this study, we first investigated the HBO1 protein expression in large numbers of tumor samples of primary breast cancer (n = 112) by IHC analysis, and showed that HBO1 was highly expressed in breast cancer (Table 1) and positively correlated with ERα (p < 0.001) and PR (p = 0.002). Moreover, HBO1 protein level correlated positively with histology grade in ER positive tumors (p = 0.016) rather than ERα negative tumors through statistical analysis. As a coactivator of the replication licensing factor Cdt1 [17], HBO1 belongs to one component of the Replication Initiation Proteins known as prereplicative complex (pre-RC) proteins. Several pre-RC proteins are over-expressed in cancer and serve as good tumor markers. And some of them, such as Cdc6 and Cdt1, are elevated by E2 treatment in breast cancer. To determine whether HBO1 was also affected by E2, quantitative real-time PCR and western blot were performed. The results suggested HBO1 was elevated after E2 treatment. Further study demonstrated the E2-induced HBO1 upregulation could be inhibited by ICI 182,780 as well as ERα siRNA.

Estrogen receptor (ER) signaling is involved in many human diseases such as breast cancer [18], lung cancer [19,20] and cardiovascular diseases [21,22]. It was reported that E2 activated the signal-transducing ERK1/2 pathway, which were critical for cell proliferation [23-25], in human mammary cancer-derived cell lines, MCF-7 and T47 D [26-28]. We explored whether ERK1/2 signaling pathway was involved in the expression of HBO1 increased by E2. The MEK1/2 inhibitor U0126 significantly inhibited the expression of HBO1 in T47 D and MCF-7 cells, suggesting that E2 increased the expression of HBO1 through the ERK1/2 signaling pathway. Since previous studies have shown that progesterone receptor activates the Src/p21ras/ERK pathway via cross-talk with estrogen receptor in breast cancer [29], the positive correlation between HBO1 protein levels and PR which we obtained in statistical analysis was reasonable.

Estrogen exposure has been regarded as high risk factor of breast cancer. It has been reported that HBO1 strongly enhanced ER-mediated transcription [9], which indicated that HBO1 might play a role in the progress of breast cancer. In this study, we proved E2 could upregulate the mRNA and protein level of HBO1. Meanwhile, knockdown of ERα with siRNA significantly inhibited the upregulation of HBO1, indicating that cross-talking was happening between ERα and HBO1 in breast cancer, the biological functions of which need to be further studied.

## Conclusion

Our data have demonstrated that the HBO1 protein levels correlated positively with ERα (p < 0.001) and PR (p = 0.002) expression in breast cancer. Further more, HBO1 protein levels correlated positively with histology grade in ERα positive tumors (p = 0.016). We also showed that the ERK1/2 signaling pathway was involved in the expression of HBO1 increased by E2. These findings suggested a potential for targeting HBO1 as a novel means of breast cancer therapy as well as a potential diagnosis marker for ERα positive breast cancer.

## Competing interests

The authors declare that they have no competing interests.

## Authors' contributions

WWZ carried out the design of the study, performed IHC, real-time PCR, drafted the manuscript. LHO performed the western blot. WYH participated in SPSS Statistical Analysis. LYH participated in IHC and IOD scoring. WWB participated in real-time PCR and cell culture. ZL performed SPSS Statistical Analysis. HY and YJW participated in IHC. SLL participated in collection of breast cancer specimens. XJJ participated in the design of the study, drafted the figure. YXJ performed the design of the study, and helped drafting the manuscript. GJX performed the collection of breast cancer specimens. All authors read and approved the final manuscript.
